# Effectiveness of implementing a locally-developed guideline for antibiotic treatment of lower urinary tract infection in adults in Thailand

**DOI:** 10.1038/s41598-023-45299-6

**Published:** 2023-10-21

**Authors:** Pruettichai Wisutep, Visanu Thamlikitkul, Rujipas Sirijatuphat

**Affiliations:** 1 Division of Infectious Diseases and Tropical Medicine, Department of Medicine, Faculty of Medicine Siriraj Hospital, Mahidol University, 2 Wanglang Road, Bangkoknoi, Bangkok, 10700 Thailand; 2 School of Medicine, Institute of Medicine, Suranaree University of Technologgy, Nakornratchasima, Thailand

**Keywords:** Infectious diseases, Antimicrobial therapy

## Abstract

Lower urinary tract infection (UTI) is still a major concern in clinical practice, but inappropriate antibiotics are commonly prescribed in Thailand. This study aimed to develop, implement, and evaluate the effectiveness of a clinical practice guideline (CPG) for antibiotic treatment of lower UTI in adults at Siriraj Hospital which is a university hospital in Thailand. This study comprised a retrospective cohort study development phase, and a prospective cohort study implementation phase. The outcomes of treatment were compared between phases. The development and implementation phases enrolled 220 and 151 patients, respectively. The CPG compliance rate was significantly increased from 17.3% during the development phase to 43.0% during the implementation phase (*p* = 0.001). The rates of fluoroquinolones and cotrimoxazole use were significantly lower during implementation than during development (*p* < 0.001 and *p* = 0.027, respectively). The rates of nitrofurantoin and fosfomycin use were significantly greater during implementation than during development (*p* = 0.009 and *p* = 0.005, respectively). The overall cure rate was not significantly different between the two study phases, but implementation group patients had significantly more unfavorable prognostic factors than development phase patients. CPG-compliance group patients had a significantly higher cure rate than CPG-non-compliance group patients (*p* = 0.011). The cost of the initial course of antibiotics per episode was significantly higher during the implementation phase because the cost of fosfomycin is more expensive and more fosfomycin was prescribed during implementation (*p* = 0.047). Implementation of the locally-developed CPG was found to be effective for increasing the appropriate use of empirical antibiotics and increasing the cure rate; however, measures to improve and reinforce CPG compliance are needed.

## Introduction

Approximately 150 million episodes of urinary tract infection (UTI) occur worldwide each year. A study conducted in the United States reported 7 million cases of acute uncomplicated UTI per year, and that the incidence of acute uncomplicated UTI is increasing annually^1^. Community-acquired lower UTI and healthcare-associated lower UTI are infections that are commonly encountered in routine clinical practice at all levels of the healthcare system from primary care to quaternary care settings^2^. Although acute lower UTI is common, prescription of inappropriate antibiotic regimens routinely occurs^3^. The emergence of multidrug-resistant (MDR) organisms continues to worsen worldwide, and is complicating decision-making regarding the selection of appropriate antimicrobial regimens^4^. Therefore, optimized clinical practice guidelines that correspond to the updated local antibiogram and local context of healthcare are necessary^5^.

The international clinical practice guidelines (CPG) for the treatment of acute uncomplicated cystitis and pyelonephritis in women that were published by the Infectious Disease Society of America (IDSA) 2011 recommend empirical antimicrobial therapy for acute uncomplicated cystitis, including nitrofurantoin, trimethoprim-sulfamethoxazole (TMP-SMX), fosfomycin trometamol, fluoroquinolones, and beta-lactam agents^2^. However, guidelines that recommend TMP-SMX or fluoroquinolones should not be applicable for Thai patients because epidemiologic study of bacteriuria at a large tertiary hospital in Thailand found the most common bacteria isolated from urine of patients with UTI to be *Escherichia coli*^6^, which is similar to the data reported from other countries^7,8^; however, the prevalence of fluoroquinolone-resistant and TMP-SMX-resistant *E. coli* at that hospital was greater than 50%^6,8^. Nevertheless, fluoroquinolone agent is still currently recommended and it remains the most commonly used agent for treatment of UTI^9^. Alternatively, the susceptibility of urinary *E. coli* isolates to nitrofurantoin and fosfomycin was reported to be greater than 90% in Thailand^6,10^, whereas the susceptibility of urinary *E. coli* isolates to amoxicillin/clavulanate was approximately 70–80%^6,10^. Since our center is responsible for managing both uncomplicated and complicated UTI, a locally-developed CPG for antibiotic treatment of lower UTI based on local data should be available.

Since no epidemiologic study of lower UTI has been conducted at our hospital, we set forth to develop, implement, and evaluate the effectiveness of a CPG for antibiotic treatment of lower UTI in adults at Siriraj Hospital which is a university hospital in Thailand.

## Patients and methods

The protocol for this study was approved by the Siriraj Institutional Review Board of the Faculty of Medicine Siriraj Hospital, Mahidol University, Bangkok, Thailand (COA no. *Si* 1029/2020). The requirement of written informed consent was waived by the Siriraj Institutional Review Board of the Faculty of Medicine Siriraj Hospital due to this being a quality of care improvement study that obtained data via retrospective medical chart review, and patient anonymity was fully preserved for all study patients. All study methods were carried out in accordance with relevant guidelines and regulations.

### Study design and data collection

This study was conducted at Siriraj Hospital, which is a 2300-bed tertiary-care university hospital, where patients with UTI can attend many different outpatient departments.

**Figure 1:**
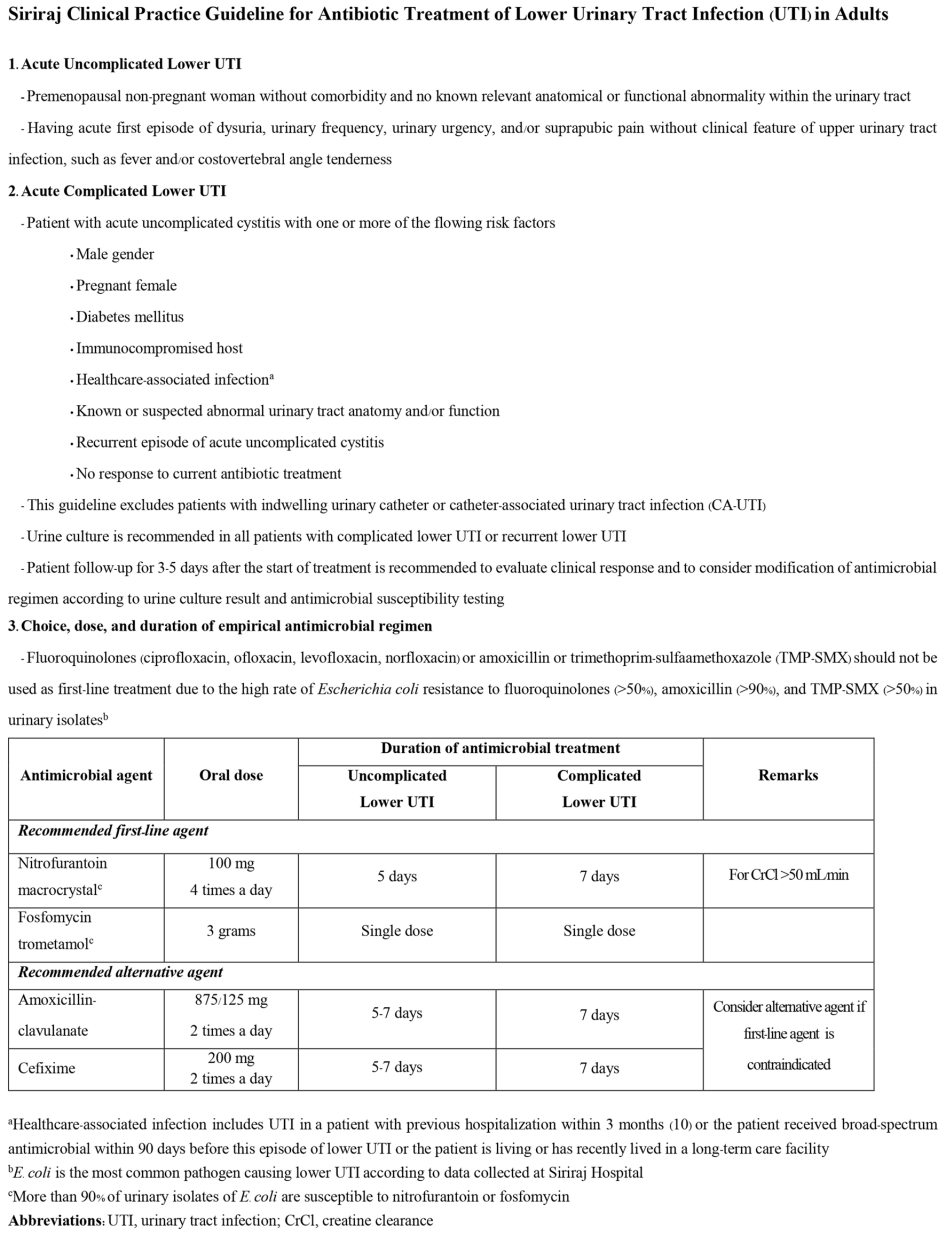
Clinical practice guideline for antibiotic treatment of lower urinary tract infection (UTI) in adults at Siriraj Hospital.

The study had two phases. The CPG development phase was an observational retrospective cohort study that reviewed the medical records of patients with UTI who attended Siriraj Hospital during 1 July 2020 to 30 April 2021 with diagnosis codes according to the International Statistical Classification of Diseases and Related Health Problems, 10th Revision (ICD-10) of N300-N309 and N390. The collected information included demographics, clinical features, causative pathogens, antibiotic treatment and treatment outcomes of patients with acute lower UTI (both uncomplicated and complicated cases) without manifestation of sepsis or acute complication of UTI who were managed as ambulatory patients. The aforementioned information combined with the recommendations of the Infectious Disease Society of America (IDSA) and the European Society for Microbiology and Infectious Diseases (ESMID)^2^, the European Association Urology guidelines on urological infections^11^, and the local antibiogram of urinary isolates from the Global Antimicrobial Resistance Surveillance System at Siriraj Hospital^6^ and the Department of Microbiology, Faculty of Medicine Siriraj Hospital, Mahidol University were used to develop the CPG for antibiotic treatment of lower UTI in adults with lower UTI at our center. The developed CPG is a simple and easy to use 2-page Thai language document (an English language translation is shown in Fig. 1). The contents of the CPG include the definition, diagnosis, investigation, and recommended antibiotic treatment of uncomplicated and complicated lower UTI in adults. This CPG is endorsed by the Division of Infectious Diseases and Tropical Medicine of the Department of Medicine, Faculty of Medicine Siriraj Hospital, Mahidol University, Bangkok, Thailand.

The CPG implementation phase was a prospective cohort study to examine the effectiveness of the implementation of our locally-developed CPG for antibiotic treatment of lower UTI in adults at Siriraj Hospital. The CPG was developed for general use among all outpatient departments that treat lower UTI at our center. We intended to start the CPG implementation phase on 1 May 2021, but the implementation phase was conducted during 1 September 2021 to 14 November 2021 due to COVID-19-related complications/restrictions. At the start, CPG implementation was performed by using multifaceted interventions including posting the CPG in outpatient care areas; disseminating the CPG as brochures, circular letters, and social media to relevant physicians; organizing conferences on the use the CPG for antibiotic treatment of lower UTI in adults; and, introducing the CPG to the relevant outpatient departments via interactive two-way communication with the responsible physicians. Our infectious disease team visited the outpatient department before the office hour started for meetings with the responsible physicians and introduced the CPG to them day by day in the weekday. Meanwhile, we made an appointment with the relevant departments to organize a brief interactive lecture. After completing the aforementioned activity, a monthly meeting with each affected outpatient department was established to remind them about and reinforce the importance of strict compliance with the CPG.

**Figure 2:**
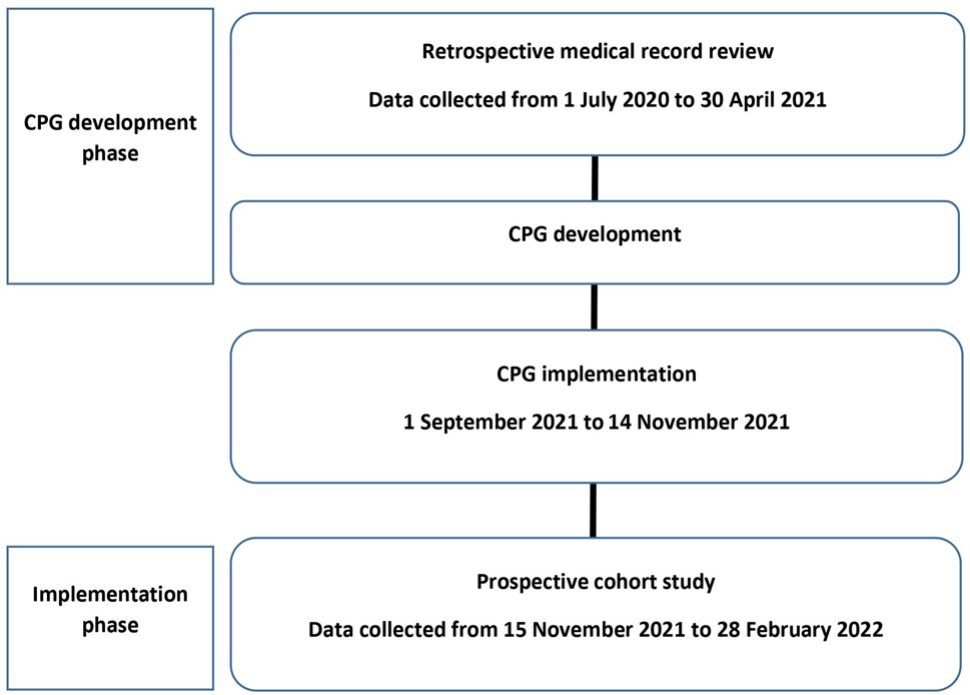
Study flow diagram.

After CPG implementation, we began to collect data of patients who met the same inclusion criteria as those enrolled during the CPG development phase. The effectiveness of this CPG was determined after 10 weeks of its implementation. Implementation phase data were collected during 15 November 2021 to 28 February 2022. The study flow diagram is shown in Fig. 2. In the case of missing clinical outcome data, patients were contacted by telephone to obtain the missing clinical outcome information.

### Study population

To be eligible for inclusion, patients had to be aged 18 years or older with a diagnosis of lower UTI that was proven or suspected to be caused by bacteria, and that was treated at an outpatient department of Siriraj Hospital. We excluded patients with lower urinary tract symptoms that were proven or suspected to be caused by non-bacteria (e.g., radiation cystitis, chemotherapy-induced cystitis), patients with indwelling urinary catheter, patients who had received hematologic or solid organ transplantation, severely immunocompromised patients, and patients without available clinical outcome data by medical record review or phone call.

No previous study in Thailand has investigated the rate of appropriate use of antibiotic for adult ambulatory patients with lower UTI. It was estimated that a sample size of 200 patients was required for the CPG development phase based on the assumption that the rate of appropriate use of antibiotic in adult ambulatory patients with lower UTI prior to implementing the use of a locally-developed CPG was 30 ± 7% with a 5% type 1 error (two-sided) and a 10% increase to compensate for cases with missing data.

We estimated the appropriateness of antimicrobial treatment for lower UTI at Siriraj Hospital (CPG compliance group) to be 30% during the CPG development phase, and that this value would increase to 50% during the CPG implementation phase. To compare the proportion for independent two groups with a power of 80% and alpha error of 5%, the sample size of patients needed for each phase with an addition of 10% in case of data missing was at least 100 patients per phase with a total of at least 200 patients in both phases.

In summary, we need at least 200 patients for retrospective cohort study for the CPG development phase and a sample size and at least 100 patients for the prospective cohort study for the CPG implementation phase.

### **Definitions**^2,7,12^

*Urinary tract infection* (UTI) was defined as the presentation of lower urinary tract symptom (s) caused by bacteria involving any part of the urinary tract confirmed by physician decision and/or evidence of pyuria (centrifuged urine leukocytes > 5 cells per high-power field).

*Lower urinary tract infection* was defined as a UTI of the urinary bladder and/or urethra, including cystitis and/or urethritis, without clinically suspected infection of the upper urinary tract.

*Upper urinary tract infection* was defined as a UTI of the kidney and/or ureter, including pyelonephritis and/or ureteritis, with usual clinical features, such as fever, costovertebral angle tenderness, and/or sepsis.

*Uncomplicated UTI* was defined as a UTI in a premenopausal woman (age ≤ 50 years) or a nonpregnant woman without comorbidities and no known relevant anatomical or functional abnormalities of the urinary tract.

*Complicated UTI* was defined as a UTI that did not satisfy the definition for uncomplicated UTI, including UTI in a male or pregnant woman, diabetes mellitus, immunocompromised host, healthcare setting, known or suspected abnormal urinary tract anatomy and/or function, and/or recurrent episode of acute uncomplicated cystitis within 3 months after the first episode of UTI.

*Immunocompromised host* was defined as a patient with neutropenia (absolute neutrophil count < 500 cells/mm^3^), immunosuppressive drug use, or acquired immunodeficiency syndrome (AIDS) with CD4 < 200 cells/mm^3^.

*Healthcare-associated infections* included a patient with previous hospitalization or who received broad-spectrum antimicrobial within 90 days before the current visit or who currently lives/recently lived in a long-term care facility.

*Clinical practice guideline (CPG) compliance* was defined as concordance between the antibiotic regimen prescribed for adult patients with a lower UTI and the antibiotic regimen recommended in the CPG.

*Clinical practice guideline (CPG) non-compliance* was defined as discordance between the antibiotic regimen prescribed for adult patients with a lower UTI and the antibiotic regimen recommended in the CPG.

*Treatment outcomes* were defined, as follows: (1) cure—resolution of all clinical features of UTI; (2) persistence—persistence of clinical features of UTI after complete treatment; (3) recurrence—occurrence of UTI within 30 days after the patient responded well to the given treatment; (4) misdiagnosis—definite diagnosis was not a UTI; and, (5) complication of UTI—progression of infection, including sepsis, upper UTI, epididymo-orchitis, requirement for hospitalization, or UTI-related death.

### Objective outcomes and statistical analysis

Due to the range of UTI antimicrobial treatment regimens, inappropriate choice, dose, or duration of treatment is common^13^. Accordingly, the establishment of and compliance with a CPG will reduce the inappropriateness of antimicrobial decision-making. The primary outcome of study was the rate of CPG compliance among the relevant physicians compared between the CPG development phase and the CPG implementation phase. The secondary outcomes were pattern of antibiotic use, outcomes of treatment, outcomes of treatment between the CPG compliance patients and the CPG non-compliance patients, and cost of treatment for adult patients with lower UTI compared between the CPG development phase and the CPG implementation phase.

Descriptive statistics were used to summarize and report patient characteristics. Comparisons of continuous data between groups were performed using unpaired Student *t*-test for normally distributed continuous data (findings reported as mean plus/minus standard deviation), and Mann–Whitney *U* test for non-normally distributed continuous data (findings reported as median and range). Comparisons of categorical data between groups were performed using chi-square test (for large sample size) or Fisher’s exact test (for small sample size) (findings reported as number and percentage). The data were analyzed using SPSS Statistics version 18.0 (SPSS, Inc, Chicago, IL, USA), A *p*-value of less than 0.05 was considered statistically significant for all tests.

### Ethical approval

The protocol for this study was approved by the Siriraj Institutional Review Board (SIRB) of the Faculty of Medicine Siriraj Hospital, Mahidol University, Bangkok, Thailand (COA no. *Si* 1029/2020). Written informed consent was not obtained from any patients in either the retrospective or prospective arms of this study because this was a quality of care improvement study that obtained data via medical chart review, and patient anonymity was fully preserved in all cases in both study phases.

## Results

There were 220 patients enrolled during the CPG development phase, and 151 patients enrolled during the CPG implementation phase. The characteristics of study patients in both phases of the study are shown in Table 1. Most patients in both phases were women (93.6% and 80.1%, respectively), and there was no significant difference in age between study phases. However, the proportion of male patients during the CPG implementation phase was significantly greater than that during the CPG development phase (19.9% *vs.* 6.4%, respectively; *p* < 0.001). Essential hypertension, diabetes mellitus, and dyslipidemia were significantly more prevalent in CPG implementation phase patients compared to their development phase counterparts. The rate of anatomical or functional abnormality of the urinary tract was comparable between the development and implementation groups (8.2% and 8.6%, respectively; *p* = 0.884). Most lower UTI cases were managed by physicians from the Internal Medicine, Urological Surgery, or Emergency Departments. The most common clinical manifestations of patients in both phases were dysuria and urinary frequency. Complicated lower UTI was the major diagnosis of patients in this study, but the prevalence of complicated lower UTI was significantly greater during the implementation phase than during the development phase (72% *vs.* 55.5%, respectively; *p* = 0.004). Previous antimicrobial exposure within 3 months before study enrollment was not significantly different between the two study phases. Urinalysis was performed in more than 90% of patients in both study phases. The rate of urine culture performed in patients during the CPG development phase was quite low (19.5%), but it significantly increased to 28.5% during the CPG implementation phase (*p* = 0.045).

**Table 1:** Patient demographic, clinical, treatment, and outcome characteristics compared between the CPG development phase group and the CPG implementation phase group (N = 371).

Characteristics	CPG development (n = 220) n (%)	CPG implementation (n = 151) n (%)	*p*-value
Gender, n (%)			
Male	14 (6.4%)	30 (19.9%)	** < 0.001**
Female	206 (93.6%)	121 (80.1%)	** < 0.001**
Age (years). mean ± SD	50.56 ± 21.37	58.46 ± 19.06	** < 0.001**
Age (years), median (range)	52 (18–97)	61 (19–93)	** < 0.001**
Comorbidity, n (%)			
Autoimmune	5 (2.3%)	5 (3.3%)	0.536
Malignancy	11 (5.0%)	9 (6.0%)	0.816
Diabetes mellitus	34 (15.5%)	45 (29.8%)	** < 0.001**
Essential hypertension	61 (27.7%)	65 (43.0%)	**0.002**
Dyslipidemia	43 (19.5%)	49 (32.5%)	**0.005**
Chronic kidney disease	15 (6.8%)	12 (7.9%)	0.681
Cirrhosis	2 (0.9%)	5 (3.3%)	0.126
Thalassemia	0 (0.0%)	2 (1.3%)	0.165
Chronic lung disease	4 (1.9%)	4 (2.6%)	0.720
Human immunodeficiency virus infection	2 (0.9%)	0 (0.0%)	0.516
Cardiovascular disease	3 (1.4%)	8 (5.3%)	0.056
Anatomical/functional abnormality of urinary tract	18 (8.2%)	13 (8.6%)	0.884
Responsible department, n (%)			
Internal medicine	57 (25.9%)	46 (30.5%)	0.336
Urological surgery	59 (26.8%)	45 (29.8%)	0.530
Emergency department	49 (22.3%)	28 (18.5%)	0.384
Gynecology	32 (14.5%)	10 (6.6%)	**0.018**
Social security clinic	13 (5.9%)	13 (8.6%)	0.317
Other	10 (4.5%)	9 (6.0%)	0.544
Clinical manifestation, n (%)			
Hematuria	73 (33.2%)	29 (19.2%)	**0.030**
Urinary frequency	105 (47.7%)	71 (47.0%)	0.893
Urinary urgency	43 (19.5%)	45 (29.8%)	**0.023**
Dysuria	170 (77.3%)	113 (74.8%)	0.588
Suprapubic pain	66 (30.0%)	36 (23.8%)	0.192
Diagnosis, n (%)			
Asymptomatic UTI	2 (0.9%)	0 (0.0%)	0.516
Uncomplicated lower UTI	81 (36.8%)	41 (27.2%)	0.052
Complicated lower UTI	122 (55.5%)	106 (70.2%)	**0.004**
Recurrent lower UTI	15 (6.8%)	4 (2.6%)	0.073
Previous antibiotic treatment within 3 months, n (%)	15 (6.8%)	11 (7.3%)	0.863
Amoxicillin	0 (0.0%)	1 (0.7%)	**0.047**
Amoxicillin/clavulanate	1 (0.5%)	0 (0.0%)	1.000
Oral third-generation cephalosporin	1 (0.5%)	2 (1.3%)	0.569
Norfloxacin	1 (0.5%)	0 (0.0%)	1.000
Ofloxacin	6 (2.7%)	1 (0.7%)	0.248
Ciprofloxacin	3 (1.4%)	7 (4.6%)	0.098
Nitrofurantoin	1 (0.5%)	0 (0.0%)	1.000
Unknown	2 (0.9%)	0 (0.0%)	0.516
Urine examination, n (%)			
No request	9 (4.1%)	14 (9.3%)	0.114
Urinalysis	211 (95.9%)	137 (90.7%)	**0.042**
Urine culture	43 (19.5%)	43 (28.5%)	**0.045**
Route of antibiotic administration, n (%)			
Oral	217 (98.6%)	151 (100%)	0.274
Parenteral	3 (1.36%)	0 (0.0%)	0.274
Type of antibiotic, n (%)			
Ceftriaxone	2 (0.9%)	0 (0.0%)	0.516
Amikacin	1 (0.5%)	0 (0.0%)	1.000
Amoxicillin	1 (0.5%)	1 (0.7%)	1.000
Amoxicillin/clavulanate	13 (5.9%)	14 (9.3%)	0.221
Oral third-generation cephalosporins	22 (10.0%)	15 (9.9%)	0.983
All fluoroquinolones (norfloxacin, ofloxacin, levofloxacin, ciprofloxacin)	128 (58.2%)	60 (39.7%)	** < 0.001**
Nitrofurantoin macrocrystal	34 (15.5%)	40 (26.5%)	**0.009**
Fosfomycin trometamol	12 (5.5%)	21 (13.9%)	**0.005**
Trimethoprim/sulfamethoxazole	7 (3.2%)	0 (0.0%)	**0.027**
Duration of the initial course of antibiotic (days), mean ± SD	5.82 ± 2.22	6.09 ± 4.02	0.407
Duration of the initial course of antibiotic (days), median (range)	5 (1–15)	5 (1–30)	0.603
CPG compliance, n (%)	38 (17.3%)	65 (43.0%)	** < 0.001**
Non-compliance	182 (82.7%)	86 (57.0%)	** < 0.001**
Inappropriate choice of antibiotic	150 (68.2%)	70 (46.4%)	** < 0.001**
Inappropriate dose of antibiotic	38 (17.3%)	22 (14.6%)	0.487
Inappropriate duration of antibiotic treatment	91 (41.4%)	47 (31.1%)	**0.045**
Treatment outcome, n (%)			
Misdiagnosis	20 (9.1%)	4 (2.6%)	**0.017**
Cure	175 (79.5%)	125 (82.2%)	0.436
Persistence	16 (7.3%)	12 (7.9%)	0.809
Recurrence	8 (3.6%)	9 (5.9%)	0.293
Complication	1 (0.5%)	1 (0.7%)	1.000
Epididymo-orchitis^a^	0 (0.0%)	1 (0.7%)	1.000
Upper UTI and sepsis^b^	1 (0.5%)	0 (0.0%)	1.000
Overall mortality	0 (0.0%)	0 (0.0%)	–
Cost of the initial course of antibiotic treatment among all study patients			
Cost of the initial course of antibiotic/patient (USD), mean ± SD	3.43 ± 6.16	4.96 ± 8.20	** < 0.001**
Cost of the initial course of antibiotic/patient (USD), median (range)	0.62 (0.26–37.00)	0.88 (0.26–48.51)	** < 0.001**
Cost of the initial course of antibiotic treatment among patients who received antibiotic according to CPG			
Cost of the initial course of antibiotic/patient (USD), mean ± SD	8.22 ± 4.18	6.28 ± 4.96	**0.047**
Cost of the initial course of antibiotic/patient (USD), median (range)	9.13 (0.88–12.07)	6.78 (0.44–12.07)	0.104

A *p*-value < 0.05 indicates statistical significance.

^a^Epididymo-orchitis found in a patient with complicated cystitis who was treated with ofloxacin 400 mg twice a day for 7 days and urine culture grew *Escherichia coli* resistant to ciprofloxacin.

^b^Upper UTI and sepsis found in a patient with complicated cystitis who was treated with ciprofloxacin 500 mg twice a day for 3 days and urine culture grew *Escherichia coli* resistant to ciprofloxacin, but hemoculture showed no growth.

*CPG* clinical practice guideline, *SD* standard deviation, *UTI* urinary tract infection, *USD* United States Dollar.

Antimicrobial prescriptions for treatment of lower UTI are shown in Table 1. In contrast to the CPG development phase, no patients received parenteral antibiotic treatment during the CPG implementation phase. Fluoroquinolones were the most commonly used agents during both study phases. The rate of fluoroquinolone (norfloxacin, ofloxacin, levofloxacin, or ciprofloxacin) use in patients during the CPG implementation phase was significantly lower than that during the CPG development phase (39.7% *vs.* 58.2%, respectively; *p* < 0.001). The use of TMP-SMX was also significantly lower during the CPG implementation phase than during the CPG development phase (0% *vs.* 3.2%, respectively; *p* = 0.027). Conversely, the use of first-line antibiotic (nitrofurantoin or fosfomycin) during the CPG implementation phase was significantly increased when compared with that during the CPG development phase (15.5% *vs.* 26.5%, respectively; *p* = 0.009 for nitrofurantoin, and 5.5% *vs.* 13.9%, respectively; *p* = 0.005 for fosfomycin). The mean and median durations of antimicrobial therapy were 5–6 days in both phases of the study, as recommended in the CPG (*p* > 0.05).

CPG compliance and treatment outcomes compared between the CPG development phase and the CPG implementation phase are shown in Table 1. The CPG compliance rate was significantly increased from 17.3% during the CPG development phase to 43.0% during the CPG implementation phase (*p* = 0.001). Inappropriate choice and inappropriate duration of antibiotic treatment during the CPG implementation phase were both significantly lower than those during the CPG development phase. The cure rate of lower UTI during the CPG implementation phase was not significantly different from that during the CPG development phase (82.2% *vs.* 79.5%, respectively; *p* = 0.436). The rates of persistent UTI, recurrent UTI, and complication of UTI were not significantly different between the CPG implementation phase and the CPG development phase. There was no UTI-related death in any patient in either phase of this study. The cost of the initial course of antibiotic was significantly higher during the CPG implementation phase than during the CPG development phase. However, the cost of the initial course of antibiotic was not significantly different between the CPG compliance subgroup of the development phase and the CPG compliance group of the implementation phase.

Although only 23% of all patients had urine culture performed, *E. coli* was still the most commonly isolated bacteria (Table 2). The *E. coli* isolates were highly susceptible to nitrofurantoin (97.1%) and fosfomycin (94.3%), moderately susceptible to coamoxiclav (71.4%), and poorly susceptible to ampicillin (22.9%), ciprofloxacin (22.8%) and TMP-SMX (45.7%). Ceftriaxone resistance was observed in 42.9% of *E. coli* isolates (Table 3). However, the antimicrobial susceptibility of fosfomycin against other bacteria was not available due to the lack of the Clinical & Laboratory Standards Institute (CLSI) standard for interpretation.

**Table 2:** Results of positive urine culture for bacteria among all patients in both study phases (N = 48).

Bacteria	n (%)
*Escherichia coli*	35 (72.9%)
*Klebsiella pneumoniae*	4 (8.33%)
Coagulase-negative staphylococci	4 (8.33%)
*Streptococcus* spp.	4 (8.33%)
*Staphylococcus aureus*	1 (0.2%)
*Proteus mirabilis*	1 (0.2%)
*Enterococcus faecalis*	1 (0.2%)

**Table 3:** Antimicrobial susceptibility profiles of the common bacteria isolated from urine culture among all patients in both study phases.

	Number	Ampicillin	Amoxicillin-clavulanate	Cefepime	Ceftriaxone	Piperacillin/tazobactam	Ertapenem	Meropenem	Imipenem	Amikacin	Ciprofloxacin	Nitrofurantoin	TMP-SMX	Fosfomycin
*Escherichia coli*	35	8	25	24	20	34	35	35	35	34	8	34	16	33
	*%*S	22.9	71.4	68.5	57.1	97.1	100	100	100	97.1	22.8	97.1	45.7	94.3
*Klebsiella pneumoniae*	4	0	2	3	3	4	4	4	4	4	3	4	3	N/A
	*%*S	0.0	50.0	75.0	75.0	100	100	100	100	100	75.0	100	75.0	N/A

*S* susceptible, *TMP-SMX* trimethoprim-sulfamethoxazole, *N/A* non-applicable.

Comparisons between the CPG compliance group and the CPG non-compliance group among all study patients during both phases are shown in Table 4. CPG non-compliance during the CPG development phase was significantly higher than CPG compliance, whereas CPG non-compliance during the CPG implementation phase was significantly lower than CPG compliance. Most characteristics of lower UTI patients in the CPG compliance group and in the CPG non-compliance group were comparable. Complicated lower UTI was the most common type of lower UTI in both groups. Most patients with lower UTI who attended the Social Security Clinic were in the CPG non-compliance group. Urine culture was performed more in the CPG compliance group than in the CPG non-compliance group (31.1% *vs.* 20.1%, respectively; *p* = 0.026). Many patients with lower UTI in the CPG non-compliance group received fluoroquinolones or amoxicillin or TMP-SMX, which are not recommended in the CPG. The cure rate of patients with lower UTI in the CPG compliance group was significantly higher than that in the CPG non-compliance group (94.2% *vs.* 75.7%, respectively; *p* < 0.001), whereas the rates of persistent infection and recurrent infection in the CPG non-compliance group were significantly higher than those in the CPG compliance group. The duration of initial antibiotic treatment in the CPG non-compliance group was significantly longer than that in CPG compliance group. The cost of the initial course of antibiotic in the CPG compliance group was significantly higher than that in CPG non-compliance group.

**Table 4:** Demographic and clinical characteristics among all patients in both study phases compared between the CPG compliance and CPG non-compliance groups (N = 371).

Characteristics	CPG compliance (n = 103) n (%)	CPG non-compliance (n = 268) n (%)	*p*-value
Period, n (%)			
CPG development group	38 (36.9%)	182 (67.9%)	** < 0.001**
CPG implementation group	65 (63.1%)	86 (32.1%)	** < 0.001**
Gender, n (%)			
Male	13 (12.6%)	31 (11.6%)	0.779
Female	90 (87.4%)	237 (88.4%)	0.779
Age (years). mean ± SD	56.63 ± 21.34	52.00 ± 20.52	**0.010**
Age (years), median (range)	59 (19–97)	55.5 (18–94)	0.880
Comorbidities, n (%)			
Autoimmune diseases	1 (1.0%)	9 (3.4%)	0.295
Malignancy	4 (3.9%)	16 (6.0%)	0.425
Diabetes mellitus	26 (25.2%)	53 (19.8%)	0.249
Essential hypertension	43 (41.7%)	83 (31.0%)	**0.050**
Dyslipidemia	32 (31.1%)	60 (22.4%)	0.083
Chronic kidney disease	11 (10.7%)	16 (6.0%)	0.118
Cirrhosis	5 (4.9%)	2 (0.7%)	**0.009**
Thalassemia	1 (1.0%)	1 (0.4%)	0.479
Chronic lung disease	2 (1.9%)	6 (2.2%)	1.000
Human immunodeficiency virus infection	0 (0.0%)	2 (0.7%)	1.000
Cardiovascular disease	6 (5.8%)	5 (1.9%)	0.079
Anatomical/functional abnormality of urinary tract	10 (9.7%)	21 (7.8%)	0.559
Responsible department, n (%)			
Internal medicine	32 (31.1%)	71 (26.5%)	0.378
Urological surgery	35 (34.0%)	69 (25.7%)	0.114
Emergency department	19 (18.4%)	58 (21.6%)	0.497
Gynecology	11 (10.7%)	31 (11.6%)	0.809
Social security clinic	2 (1.9%)	24 (9.0%)	**0.018**
Other	4 (3.9%)	15 (5.5%)	0.503
Clinical manifestations, n (%)			
Hematuria	20 (19.4%)	82 (30.6%)	**0.031**
Urinary frequency	55 (53.4%)	121 (45.1%)	0.154
Urinary urgency	35 (34.0%)	53 (19.8%)	**0.004**
Dysuria	77 (74.8%)	206 (76.9%)	0.669
Suprapubic pain	22 (21.4%)	80 (29.9%)	0.101
Diagnosis, n (%)			
Asymptomatic UTI	1 (1.0%)	1 (0.4%)	0.479
Uncomplicated lower UTI	30 (29.1%)	92 (34.3%)	0.339
Complicated lower UTI	63 (61.2%)	165 (61.6%)	0.943
Recurrent lower UTI	9 (8.7%)	10 (3.7%)	**0.050**
Previous antibiotic treatment within 3 months, n (%)	12 (11.7%)	14 (5.2%)	**0.030**
Amoxicillin	0 (0.0%)	1 (0.4%)	1.000
Amoxicillin/clavulanate	1 (1.0%)	0 (0.0%)	0.278
Oral third-generation cephalosporin	2 (1.9%)	1 (0.4%)	0.188
Norfloxacin	0 (0.0%)	1 (0.4%)	1.000
Ofloxacin	3 (2. 9%)	4 (1.5%)	0.402
Ciprofloxacin	5 (4.9%)	5 (1.9%)	0.148
Nitrofurantoin	1 (1.0%)	0 (0.0%)	0.278
Unknown	0 (0.0%)	2 (0.7%)	1.000
Urine examination, n (%)			
No request	3 (2.9%)	18 (6.7%)	0.211
Urinalysis	100 (97.1%)	248 (92.5%)	0.104
Urine culture	32 (31.1%)	54 (20.1%)	**0.026**
Route of antibiotic administration, n (%)			
Oral	103 (100%)	265 (98.9%)	0.563
Parenteral	0 (0.0%)	3 (1.1%)	0.563
Type of antibiotics, n (%)			
Ceftriaxone	0 (0.0%)	2 (0.7%)	1.000
Amikacin	0 (0.0%)	1 (0.4%)	1.000
Amoxicillin	0 (0.0%)	2 (0.7%)	1.000
Amoxicillin/clavulanate	23 (22.3%)	4 (1.5%)	** < 0.001**
Oral third generation cephalosporins	13 (12.6%)	24 (9.0%)	0.291
All fluoroquinolones (norfloxacin, ofloxacin, levofloxacin, ciprofloxacin)	0 (0.0%)	188 (70.1%)	** < 0.001**
Nitrofurantoin macrocrystal	34 (33.0%)	40 (14.9%)	** < 0.001**
Fosfomycin trometamol	33 (32.0%)	0 (0.0%)	** < 0.001**
Trimethoprim/sulfamethoxazole	0 (0.0%)	7 (2.6%)	0.197
Duration of the initial course of antibiotic (days), mean ± SD	4.69 ± 2.6	6.41 ± 3.09	** < 0.001**
Duration of the initial course of antibiotic (days), median (range)	5 (1–7)	5 (3–30)	**0.002**
Outcomes, n (%)			
Misdiagnosis	4 (3.9%)	20 (7.5%)	0.209
Cure	97 (94.2%)	203 (75.7%)	** < 0.001**
Persistence	2 (1.9%)	26 (9.7%)	**0.011**
Recurrence	0 (0.0%)	17 (6.3%)	**0.005**
Complication	0 (0.0%)	2 (0.7%)	1.000
Epididymo-orchitis	0 (0.0%)	1 (0.4%)	1.000
Upper UTI and sepsis	0 (0.0%)	1 (0.4%)	1.000
Overall mortality	0 (0.0%)	0 (0.0%)	1.000
Cost of the initial course of antibiotic/patient (USD), mean ± SD	7.01 ± 4.75	2.91 ± 7.51	** < 0.001**
Cost of the initial course of antibiotic/patient (USD), median (range)	6.78 (0.88–12.07)	0.62 (0.26–48.51)	** < 0.001**

A *p*-value < 0.05 indicates statistical significance.

*CPG* clinical practice guideline, *SD* standard deviation, *UTI* urinary tract infection, *USD* United States Dollar.

Comparisons between the CPG compliance group and CPG non-compliance group among the 151 patients enrolled in the CPG implementation phase are shown in Table 5. Gender, age, and comorbidities were non-significantly different between groups. Complicated lower UTI and recurrent lower UTI were observed in 67.7% *vs.* 72.1% and 4.6% *vs.* 1.2% of patients in the CPG compliance group and CPG non-compliance group respectively while urine culture was performed in only 29.2% *vs.* 27.9% of the patients with lower UTI in the CPG compliance group and CPG non-compliance group respectively. The patients with lower UTI who attended the Social Security Clinic significantly more often received empirical antibiotics that are not recommended in the CPG (*p* = 0.007). Many patients with lower UTI in the CPG non-compliance group still received fluoroquinolones, which are not recommended in the CPG, whereas a smaller number of patients with UTI in the CPG non-compliance group received nitrofurantoin, which is recommended in the CPG. The cure rate of patients with lower UTI in the CPG compliance group was significantly greater than that in the CPG non-compliance group (96.9% *vs.* 72.1%, respectively; *p* < 0.001), whereas the rates of persistent infection and recurrent infection in the CPG non-compliance group were significantly greater than those in the CPG compliance group. The duration of antibiotic therapy for lower UTI in the CPG non-compliance group was significantly longer than that in the CPG compliance group. Although the mean cost of the initial course of antibiotics for lower UTI patients in the CPG compliance group was not significantly different from that in the CPG non-compliance group, the median cost of the initial course of antibiotics for lower UTI patients in the CPG compliance group was significantly higher than that in the CPG non-compliance group.

**Table 5:** Demographic and clinical characteristics of patients included in the implementation phase compared between the CPG compliance and CPG non-compliance groups (N = 151).

Characteristics	CPG compliance (n = 65) n (%)	CPG non-compliance (n = 86) n (%)	*p*-value
Gender			0.430
Male	11 (16.9%)	19 22.1%)	–
Female	54 (83.1%)	67 (77.9%)	–
Age (years). mean ± SD	59.82 ± 20.08	57.43 ± 18.30	0.448
Age (years), median (range)	64 (19–93)	60 (22–91)	0.355
Comorbidities, n (%)			
Autoimmune diseases	1 (1.5%)	4 (4.7%)	0.391
Malignancy	3 (4.6%)	6 (7.0%)	0.733
Diabetes mellitus	21 (32.3%)	24 (27.9%)	0.558
Essential hypertension	30 (46.2%)	35 (40.7%)	0.503
Dyslipidemia	25 (38.5%)	24 (27.9%)	0.170
Chronic kidney disease	8 (12.3%)	4 (4.7%)	0.085
Cirrhosis	3 (4.6%)	2 (2.3%)	0.652
Thalassemia	1 (1.5%)	1 (1.2%)	1.000
Chronic lung disease	1 (1.5%)	3 (3.5%)	0.635
Cardiovascular disease	5 (7.7%)	3 (3.5%)	0.291
Anatomical/functional abnormality of urinary tract	7 (10.8%)	6 (7.0%)	0.411
Responsible department, n (%)			
Internal medicine	24 (36.9%)	22 (25.6%)	0.134
Urological surgery	20 (30.8%)	25 (29.1%)	0.821
Emergency department	12 (18.5%)	16 (18.6%)	0.982
Gynecology	6 (9.2%)	4 (4.7%)	0.262
Social security clinic	1 (1.5%)	12 (14.0%)	**0.007**
Other	2 (3.1%)	7 (8.1%)	0.193
Clinical manifestations, n (%)			
Hematuria	10 (15.4%)	19 (22.1%)	0.300
Urinary frequency	34 (52.3%)	37 (4.3%)	0.258
Urinary urgency	27 (41.5%)	18 (20.9%)	**0.006**
Dysuria	47 (72.3%)	66 (76.7%)	0.534
Suprapubic pain	18 (27.7%)	18 (20.9%)	0.334
Diagnosis, n (%)			
Uncomplicated lower UTI	18 (27.7%)	23 (26.7%)	0.897
Complicated lower UTI	44 (67.7%)	62 (72.1%)	0.558
Recurrent lower UTI	3 (4.6%)	1 (1.2%)	0.315
Previous antibiotic treatment within 3 months	7 (10.8%)	4 (4.7%)	0.208
Amoxicillin	0 (0.0%)	1 (1.2%)	1.000
Oral third generation cephalosporin	2 (3.1%)	0 (0.0%)	0.184
Ofloxacin	1 (1.5%)	0 (0.0%)	0.430
Ciprofloxacin	4 (6.2%)	3 (3.5%)	0.464
Urine examination, n (%)			
No request	2 (3.1%)	10 (11.6%)	0.054
Urinalysis	63 (96.9%)	74 (86.0%)	**0.023**
Urine culture	19 (29.2%)	24 (27.9%)	0.858
Route of antibiotic administration, n (%)			
Oral	65 (100%)	86 (100%)	1.000
Type of antibiotics, n (%)			
Amoxicillin	0 (0.0%)	1 (1.2%)	1.000
Amoxicillin/clavulanate	11 (16.9%)	3 (3.5%)	**0.005**
Oral third-generation cephalosporins	6 (9.2%)	9 (10.5%)	1.000
All fluoroquinolones (norfloxacin, ofloxacin, levofloxacin, ciprofloxacin)	0 (0.0%)	60 (69.8%)	** < 0.001**
Nitrofurantoin macrocrystal	27 (41.5%)	13 (15.1%)	** < 0.001**
Fosfomycin trometamol	21 (32.3%)	0 (0.0%)	** < 0.001**
Duration of the first course of antibiotic (days), mean ± SD	4.57 ± 2.61	7.24 ± 4.50	** < 0.001**
Duration of the first course of antibiotic (days), median (range)	5 (1–7)	7 (3–30)	0.213
Outcomes, n (%)			
Misdiagnosis	1 (1.5%)	3 (3.5%)	0.635
Cure	63 (96.9%)	62 (72.1%)	** < 0.001**
Persistence	1 (1.5%)	11 (12.8%)	**0.011**
Recurrence	0 (0.0%)	9 (10.5%)	**0.010**
Complication	0 (0.0%)	1 (1.2%)	1.000
Epididymo-orchitis	0 (0.0%)	1 (1.2%)	1.000
Overall mortality	0 (0.0%)	0 (0.0%)	–
Cost of the initial course of antibiotic/patient (USD), mean ± SD	6.30 ± 4.96	3.95 ± 9.89	0.082
Cost of the initial course of antibiotic/patient (USD), median (range)	6.78 (0.88–12.07)	0.62 (0.26–48.51)	** < 0.001**

A *p*-value < 0.05 indicates statistical significance.

*CPG* clinical practice guideline, *SD* standard deviation, *UTI* urinary tract infection, *USD* United States Dollar.

## Discussion

The objective of this study was to develop and implement a CPG for antibiotic treatment of lower UTI in adults because we observed that many patients with lower UTI received fluoroquinolones and some patients received amoxicillin or TMP-SMX, and the international guidelines for antibiotic treatment of lower UTI in adults still recommend TMP-SMX as one of the first-line antibiotics^2,7,14,15^. The reason that this is a problem in a Thai setting is because the annual antimicrobial susceptibility results of bacteria isolated from patients at Siriraj Hospital showed more than 80% of urinary isolates of *E. coli* to be resistant to ampicillin, more than 50% of urinary isolates of *E. coli* to be resistant to fluoroquinolones and TMP-SMX, and nearly 50% of urinary isolates of *E. coli* to be resistant to ceftriaxone. To develop a CPG for antibiotic treatment of lower UTI in adults at Siriraj Hospital, we combined patient demographic information, clinical features, causative pathogens and their antimicrobial susceptibility results, antibiotic treatment, and treatment outcomes of patients with lower UTI at our center during 1 July 2020 to 30 April 2021 with annual antimicrobial susceptibility results of urinary isolates of *E. coli* and some appropriate recommendations from the international guidelines. The findings of this study resulted in nitrofurantoin and fosfomycin being recommended in our CPG as the first-line antibiotics for lower UTI in adults at Siriraj Hospital because more than 90% of urinary isolates of *E. coli* were susceptible to nitrofurantoin and fosfomycin, including many *E. coli* isolates that were resistant to amoxicillin, fluoroquinolones, TMP-SMX, and ceftriaxone. Information that was retrospectively collected during the 1 July 2020 to 30 April 2021 CPG development phase was used as baseline data that was subsequently compared with the information collected during the 15 November 2021 to 28 February 2022 CPG implementation phase to evaluate the effectiveness of the CPG. Due to the nature of the pre- and post-CPG implementation study, some baseline characteristics of study patients between the prospective phase and the retrospective phase were different. However, the primary outcome (rate of CPG compliance) and some secondary outcomes (i.e., pattern of antibiotic use and cost of treatment) of the study should not be influenced by these baseline parameters but should be impacted by the CPG implementation.

Our locally-developed CPG for antibiotic treatment of lower UTI in adults was implemented at Siriraj Hospital using multifaceted interventions because this type of implementation was reported to be effective measure for guideline implementation^9,16–19^. Although the CPG compliance rate was significantly increased from 17.3% during the CPG development phase to 43.0% during CPG implementation phase (*p* = 0.001), and the rate of inappropriate choice (fluoroquinolones) of antibiotic treatment during the CPG implementation phase was significantly lower than the rate during the CPG development phase, the rate of CPG compliance of 43% is still too low, and fluoroquinolones were still the most commonly prescribed agents to treat lower UTI during the CPG implementation phase. Therefore, additional measures need to be developed and implemented to inform, remind, and reinforce the use CPG among clinicians, especially those working in our center’s Social Security Clinic.

Even though the rate of urine culture was significantly increased from 19.5% during the CPG development phase to 28.5% during the CPG implementation phase, the latter urine culture rate was low because our CPG recommends urine culture in patients with complicated UTI or recurrent UTI, and 72.8% of patients during the CPG implementation phase had complicated UTI or recurrent UTI. Reinforcement of the need to order a urine culture in patients with complicated UTI or recurrent UTI is, therefore, needed.

The cure rate of lower UTI during the CPG implementation phase was non-significantly different from the cure rate observed during the CPG development phase. This finding is likely explained by important differences in some important characteristics between the two study phases. The proportions of male gender, of patients with comorbidities, and of patients with complicated UTI were all significantly greater during the CPG implementation phase than during the CPG development phase, and those patients were reported to have a poorer response to treatment^20–22^. However, the cure rate of the lower UTI patients who received treatment that complied with the treatment recommendations in the CPG was significantly higher than the cure rate in patients who did not receive antibiotic that complied with the CPG during both the CPG development phase and the CPG implementation phase. This remarkable finding strongly indicates that despite the low compliance rate of the CPG and a higher prevalence of complicated UTIs in the prospective period, compliance with the developed CPG was associated with improved clinical outcomes compared to the outcomes of patients who did not receive care that complied with the CPG, and it appears to be a major justification for using the CPG for patient care.

The cost of the initial course of antibiotic was more expensive during the CPG implementation phase than during the CPG development phase because the CPG recommends fosfomycin as one of the first-line agents for lower UTI, and the cost of fosfomycin is much higher than the inappropriately used agents, such as fluoroquinolones, amoxicillin, and TMP-SMX. Given our finding that persistent infection and recurrent infection were both significantly more prevalent in the CPG non-compliance group during the CPG development phase than in the CPG compliance group during the CPG implementation phase, patients receiving inappropriate antibiotic would need to return to hospital and be prescribed a second course of antibiotics that would normally be more expensive than the first course of antibiotics. Therefore, the total cost of curing a patient in the CPG non-compliance group might be similar to or even more expensive than the cost of curing a patient in the CPG compliance group.

The healthcare scheme in Thailand may influenced the selection of drugs. There are 3 main types of the healthcare scheme in Thailand i.e. Government Enterprise Officer coverage, Universal Coverage Scheme, and Social Security Scheme. For Government Enterprise Officer coverage, this scheme provide most of expenses including the non-essential drug list in Thailand (e.g., fosfomycin is in this list), so the government officers can access the non-essential drug list by free. For Universal Coverage Scheme and Social Security Scheme, the patients in these schemes cannot access the medications in the non-essential drug list, they have to pay for those drugs. As aforementioned, the use of fosfomycin for treating lower UTI may be reduced for patients under the Universal Coverage Scheme and the Social Security Scheme. Because fosfomycin is a non-essential drug in Thailand that has a barrier to access under the Universal Coverage Scheme and Social Security Scheme, we listed nitrofurantoin in Thailand's essential drug list as the first line in our CPG.

### Strengths and limitations

This study has several notable strengths. First, the CPG was locally-developed using data from hundreds of UTI patients who were treated at Siriraj Hospital, and using data specific to the antibiotic susceptibility results of locally obtained urinary *E. coli* isolates. Second, CPG implementation was performed using multifaceted interventions. Third, CPG compliance, clinical outcomes, and cost of treatment were compared between the CPG development phase and the CPG implementation phase. Fourth and last, in order to promote the appropriate use of antibiotics, this study showed the value of adopting locally-developed CPG. The methods used for implementation were straightforward but effective enough, and they could be used at all levels of healthcare systems by adapting them to the local context, not only for the UTI but for all other infectious diseases as well.

This study also has some mentionable limitations. First, this was a single-center retrospective study during CPG development phase; however, we set forth to develop a CPG for our center, and the retrospective component of this study was used to compare with the prospective component of this study. Second, CPG compliance during the CPG implementation phase was rather low, so efforts to improve awareness of and compliance with this CPG are needed. Third and finally, there is a scarcity of data regarding the factors associated with CPG non-compliance, so further study on the factors associated with CPG non-compliance is needed to improve the CPG implementation process for this and future CPGs.

## Conclusions

Implementation of the locally-developed CPG was found to be effective for increasing the appropriate use of empirical antibiotics and increasing the cure rate among adult ambulatory patients who attend outpatient clinics at Siriraj Hospital for diagnosis and treatment of lower UTI. However, the level of compliance with the CPG during the implementation phase of this study is still unsatisfactory. Therefore, improved measures are needed to enhance CPG awareness and improve CPG compliance—both of which will improve patient outcomes.

## Data Availability

The datasets used and/or analysed during the current study are available from the corresponding author on reasonable request.
